# Neuroblastoma presenting with symptoms of epidural compression at birth: a case report

**DOI:** 10.1186/s13052-016-0263-6

**Published:** 2016-05-21

**Authors:** Chiara Suffia, Stefania Sorrentino, Simona Vetrella, Delfina Bifano, Marilina Nantron, Bruno De Bernardi, Carlo Gandolfo

**Affiliations:** Departments of Hematology-Oncology, Istituto Giannina Gaslini, Genoa, Italy; Department of Hematology-Oncology, Santobono-Pausilipon Children’s Hospital, Naples, Italy; Department of anatomopathology, Santobono-Pausilipon Children’s Hospital, Naples, Italy; Pediatric Interventional Radiology and Neuroradiology Unit, Istituto Giannina Gaslini, Genoa, Italy

**Keywords:** Neuroblastoma, Newborn, Epidural compression, Late sequelae, Case report

## Abstract

**Background:**

Five to 10 % of children with neuroblastoma present with symptoms of epidural compression (EC). More than half these patients are diagnosed in the first year of life. The case of a neuroblastoma presenting symptoms of EC at birth is exceptional and deserves to be reported.

**Case presentation:**

We describe a case of female born at the 36^th^ week of pregnancy by caesarian section decided following ultrasonographic discovery of oligohydramnios. At birth, she was noted to have motor deficit involving both legs and continuous urinary dripping. These symptoms were found to be secondary to a paraspinal neuroblastoma infiltrating the spinal canal. Tumor responded well to chemotherapy, but neurologic deficit only slightly improved and bladder dysfunction remained unchanged. At 2 years of age, patient is able to walk with help of leg orthoses, suffers chronic constipation requiring daily medications, and has neurologic bladder necessitating multiple daily catheterizations.

**Conclusions:**

The finding of a newborn presenting with symptoms of EC secondary to a neuroblastoma invading the spinal canal is quite uncommon. The case described herewith confirms that these rare patients have an excellent survival probability, but almost always develop severe functional sequelae.

## Background

Neuroblastoma accounts for 8–10 % of pediatric cancers [1]. Five to 10 % of children with neuroblastoma present with symptoms of epidural compression (EC) [2, 3]. More than half these patients are diagnosed in the first year of life [4]. These young patients have a high survival probability, but almost all develop severe sequelae [4, 5]. The discovery of a neuroblastoma associated with EC at birth is a rare event [6], as witnessed by the fact that only 0.2 % of all diagnoses of this tumour were registered by the Italian Association of Hematology and Oncology (AIEOP) Neuroblastoma Registry over a period of 10 years [7]. The literature concerning these patients is limited to a small number of mostly single-case reports [6, 8–16]. We describe herewith a newborn whose delivery was anticipated to the 36^th^ week of pregnancy for olygohydramnios and was found to have motor deficit and bladder dysfunction at birth secondary to an abdominal neuroblastoma.

## Case presentation

**Fig. 1:**
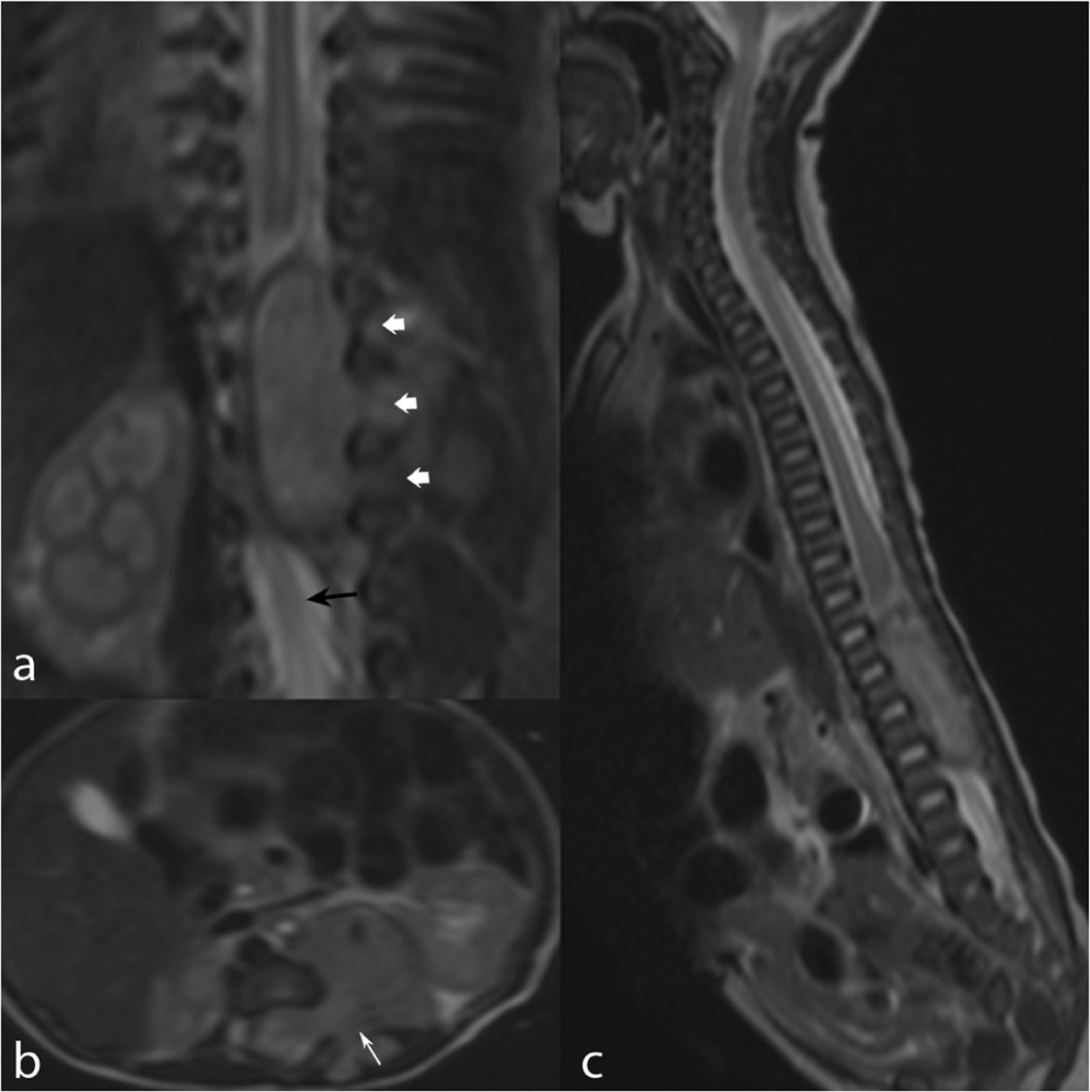
MRI at diagnosis. Three planar T2WI (**a**, **b**, **c**). Retroperitoneal neuroblastoma with intraspinal invasion. Left-sided large mass with dumbbell extension through widened neural foramina (**a** and **b**, *white arrows*) and marked compression and dislocation of the cauda (**a**, *black arrow*)

**Fig. 2:**
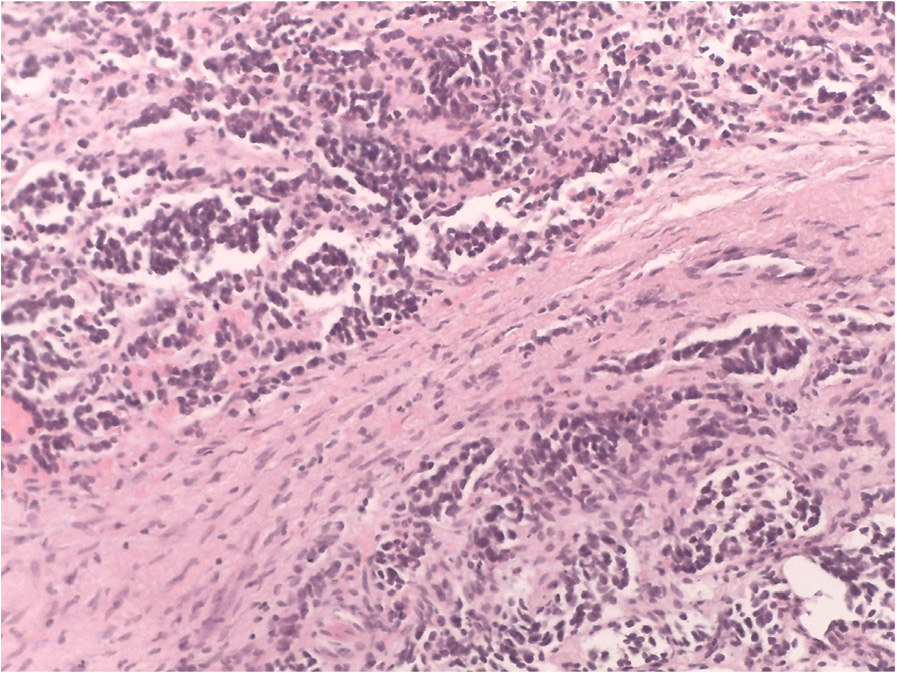
Histological pattern. Nests of undifferentiated round blue cells, forming vague Homer-Wright rosettes. EE 200×

A female was delivered on February 28, 2014, by caesarian section performed on the 36^th^week of pregnancy following the ultrasonographic discovery of severe oligohydramnios. The examination was read negative for the presence of abdominal masses and did not document reduced foetal movements. Birth weight was 2300 grams. Apgar score was within normal limits. Physical examination in the first day of life showed hypotonia and no motility of legs, and distended bladder with continuous urinary dripping requiring catheterization. Spinal Magnetic resonance imaging (MRI) was performed on the 2nd day of life and showed a solid retroperitoneal mass, 4×1.5 cm in diameter, entering the spinal canal through the foramina from D11-12 until L2-L3 (Fig. 1). This finding, together with the elevated excretion of urinary vanillylmandelic acid, was suggestive of a peripheral neuroblastic tumor (PNT). Following multidisciplinary discussion and achievement of family consent, decision was taken to avoid the neurosurgical approach in favor of chemotherapy. Based on the guidelines of the International Society of Pediatric Oncology Europe Neuroblastoma (SIOPEN) for symptomatic PNTs presenting with EC [17], an association of carboplatin and etoposide was initiated on day 6 from birth (Table 1). Tru-cut biopsy of the extraspinal tumor was performed on day 21 of life. The histopathologic examination revealed a Schwannian stroma-poor, poorly differentiated neuroblastoma composed of undifferentiated, primitive-appearing, round blue cells in a nesting pattern (Fig. 2). Focally, these cells were embedded in a clearly recognizable neuropil [18]. Immunoreactivity for thyrosinase, CD56, synaptophysin, chromogranin, neuron-specific enolase and negativity for CD99, actin, CD45, WT1, CK, and CD34 were read negative. Due to the scarcity of the available tissue no biologic studies could be performed. Two bone marrow aspirates were found free of tumor cells. On day 33, meta-iodobenzylguanidine (MIBG) scintigraphy showed intense positivity at site of the tumor and no metastatic lesions. Tumor extension was thus defined as International Neuroblastoma Staging System (INSS) stage 3 [19]. At the MRI performed after 4 chemotherapy courses (on day 103), the intraspinal tumor appeared markedly decreased. At this time, the MIBG-positive lesion had disappeared. Neurologically, the motor deficit had progressively decreased, more for the left leg, while bladder dysfunction was unchanged. Based on clinical and imaging responses, antitumor therapy was stopped. Patient was included in an intensive physiotherapy program aiming to further improve motor deficit and flexibility of lower limbs.

**Table 1:** Drug dosages and schedules

Drug Associations	Drugs	Days
Carbo/VP	• Carboplatin 4 mg/kg	1–3
	• Etoposide 3,34 mg/kg	1–3, repeated after 3 weeks
CADO	• Cyclophosphamide 10 mg/kg	1–5
	• Doxorubicin 1 mg/kg	4–5
	• Vincristine 0.05 mg/kg	1 and 5, repeated after 4 weeks

**Fig. 3:**
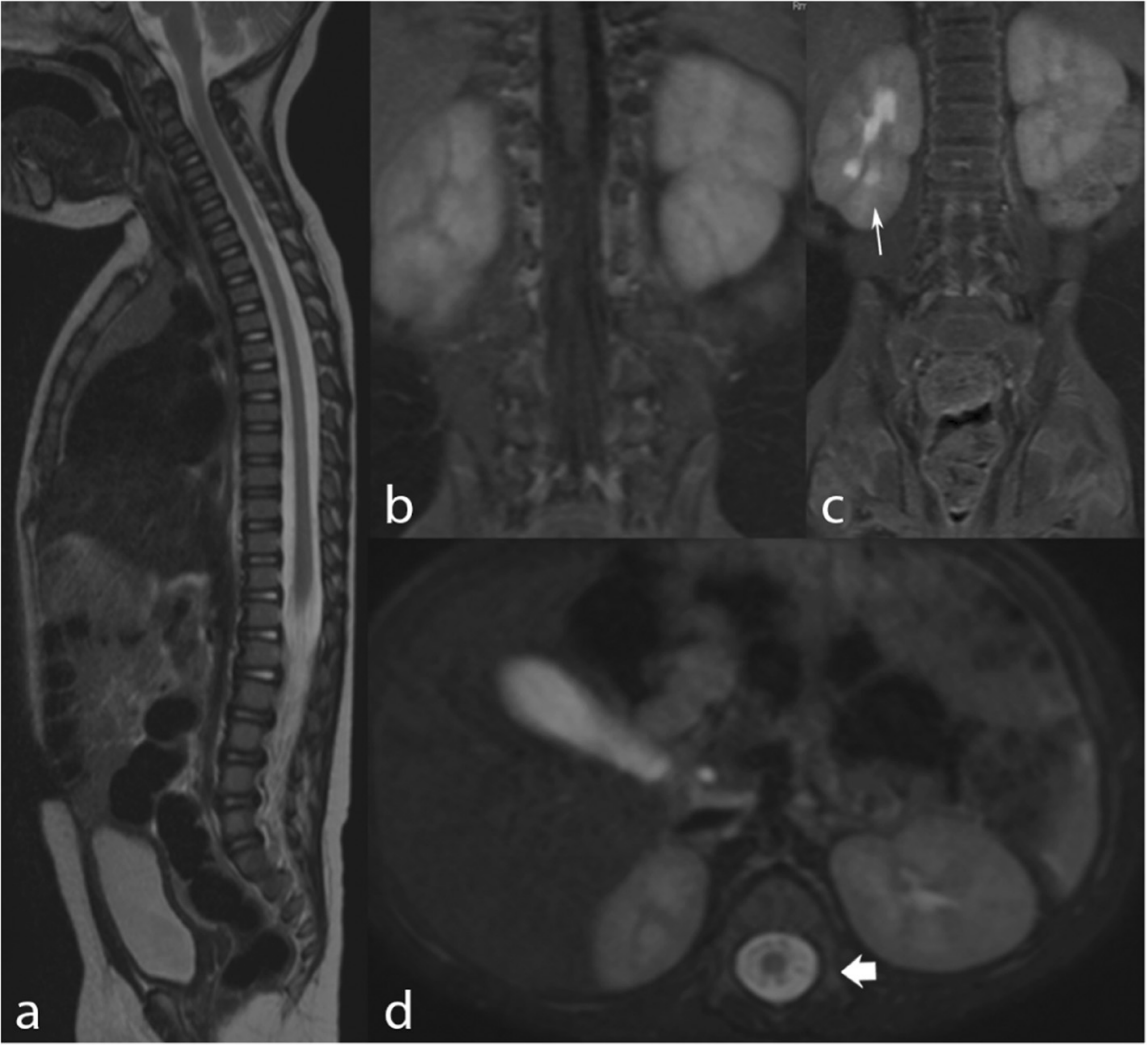
MRI after conclusion of chemotherapy. Three planar T2WI (**a**, **c**, **d**) and coronal T1WI (**b**). The exam shows complete regression of the tumor at both intra- and extraspinal compartments. Mild pyelectasia was detected in the right kidney

At the follow-up evaluations, scheduled at 4–6 months intervals, spinal MRI showed complete regression of both intra- end extraspinal tumors (Fig. 3) with normalization of catecholamine metabolites urinary excretion. Patient experienced further neurologic improvement making her able to assume the upright posture.

Presently, at 2 years of age, patient is able to walk with help of leg orthoses to correct feet abnormalities (equinovarus supinatus feet). In addition, she complains chronic constipation requiring daily medication, and neurogenic bladder necessitating multiple daily catheterizations and antibiotic prophylaxis.

## Discussion

Children diagnosed with neuroblastoma presenting with symptoms of EC have an excellent outcome in reason of the favourable location of the primary tumour and the rare metastatic spread [1–3]. Children at this age usually have favorable biologic features (in particular, a single copy of *MYCN* oncogene, and no abnormal *1p* and *11q* chromosomes) [17, 20], and are thus excellent candidates to cure. Although adequate studies could not be performed due to the insufficient amount of tumour tissue available, it is conceivable that the tumour of the patient had normal biologic profile. On the other hand, the majority of these patients develop long-term sequelae and this finding did not decrease since the first reported series, back more than two decades ago [2]. The risk of developing sequelae was greater in younger children [4, 5]. The occurrence of symptoms of EC in newborns with neuroblastoma has been occasionally described [6, 8–16]. Of note, all the reported cases have developed mostly severe sequelae, with exception of two patients, both described in 2008, surviving without significant late effects [15, 16]. Both were delivered prematurely following late pregnancy ultrasound detecting an extraspinal tumor mass.

Also in our patient anticipating delivery was decided on the basis of late pregnancy ultrasound, that did discover the presence of oligohydramnios, but missed an abdominal tumor mass. Two main reasons may account for the failure: i) an abdominal mass may be too small at the 36th week of pregnancy to be detected by ultrasound, and ii) at this time of pregnancy the amniotic fluid is reduced and the mineralization of the fetal skeleton is increased, making it difficult to assess the paravertebral regions, especially when the fetus’ back is lying opposite to the ultrasound beam.

Despite early delivery, prompt diagnostic work-up and initiation of chemotherapy, the neurologic deficits only partly regressed leading to the establishment of definitive neurologic and functional sequelae. Therefore, the clinical course of our patient did not replicate what occurred to the two patients described in 2008, both surviving free of sequelae. The fact that no further case with similar favorable course has been reported since and the unfavorable course of our patient, suggest that anticipating delivery might be not always sufficient to prohibit the development of late sequelae. Despite that, it is conceivable that greater awareness of sonologists might increase the possibility to recognize a paravertebral mass in a fetus. When this occurs, MRI should be promptly performed to document the possible invasion of the spinal canal. With these information in hands, the interdisciplinary team may share with the family the decision to anticipate delivery. In the case symptoms of EC are evident at birth, prompt initiation of chemotherapy may translate into lesser risk of long-term sequelae and better quality of life for some of these patients.

## Conclusions

We describe a newborn prematurely delivered because of oligohydramnios, who had symptoms of EC at birth secondary to a paraspinal neuroblastoma. Despite prompt initiation of therapy, the neurologic deficits only partly regressed leading to the establishment of severe definitive physical disabilities. The unfavorable functional outcome of our patient contrasts with the successful course of two previous cases delivered prematurely. This difference in outcome suggests that anticipating delivery may not exempt from development of sequelae. However, accurate ultrasonography in late pregnancy is to be supported, as it may increase the chance to detect an abdominal tumor and this may lead to timely therapy and better functional outcome in some of these patients.

### Ethics approval and consent to participate

Not applicable.

## Consent to publish

Written informed consent was obtained from the patient’s parents for publication of this Case report and any accompanying images. A copy of the written consent form is available for review by the Editor-in-Chief of this journal.

## Abbreviation

AIEOP: Associazione Italiana Ematologia Oncologia Pediatrica
EC: epidural compression
INSS: International Neuroblastoma Staging System
MIBG: meta-iodobenzylguanidine
MRI: magnetic resonance imaging
PNT: peripheral neuroblastic tumor
SIOPEN: International Society of Pediatric Oncology Europe Neuroblastoma
T1WI: weighted image
T2WI: weighted image
